# Involvement of the Inflammasome and Th17 Cells in Skin Lesions of Human Cutaneous Leishmaniasis Caused by *Leishmania* (*Viannia*) *panamensis*

**DOI:** 10.1155/2020/9278931

**Published:** 2020-10-27

**Authors:** K. Gonzalez, J. E. Calzada, C. E. P. Corbett, A. Saldaña, M. D. Laurenti

**Affiliations:** ^1^Departamento de Parasitología Molecular, Instituto Conmemorativo Gorgas de Estudios de la Salud, Ave. Justo Arosemena, Calidonia, 0816-02593 Panama City, Panama; ^2^Departamento de Patología, Laboratorio de Patologia de Moléstias Infecciosas, Faculdade de Medicina, Universidade de São Paulo, Ave. Doutor Arnaldo 455, 01246-903 São Paulo Cerqueira César, Brazil; ^3^Facultad de Medicina Veterinaria, Universidad de Panamá, Campus Harmodio Arias Madrid, Av. Juan Pablo II, Albrook, Panama City, Panama; ^4^Centro de Investigación y Diagnóstico de Enfermedades Parasitarias, Facultad de Medicina, Universidad de Panamá, Ave. Octavio Méndez Pereira, Panama City, Panama

## Abstract

Localized cutaneous leishmaniasis (LCL) caused by *Leishmania* (*Viannia*) *panamensis* is an endemic disease in Panama. This condition causes ulcerated skin lesions characterized by a mixed Th1/Th2 immune response that is responsible for disease pathology. However, the maintenance of the *in situ* inflammatory process involves other elements, such as Th17 and inflammasome responses. Although these processes are associated with parasite elimination, their role in the increase in disease pathology cannot be discarded. Thus, the role in *Leishmania* infection is still unclear. In this sense, the present study aimed at characterizing the Th17 and inflammasome responses in the skin lesions of patients with LCL caused by *L.* (*V.*) *panamensis* to help elucidate the pathogenesis of this disease in Panama. Th17 and inflammasome responses were evaluated by immunohistochemistry (IHQ) in 46 skin biopsies from patients with LCL caused by *L.* (*V.*) *panamensis*. The Th17 immune response was assessed using CD3, CD4, RoR*γ*t, IL-17, IL-6, IL-23, and TGF-*β*1 antibodies, and the inflammasome response was assessed by IL-1*β*, IL-18, and caspase-1 antibodies. The presence of the Th17 and inflammasome responses was evidenced by a positive reaction for all immunological markers in the skin lesions. An inverse correlation between the density of amastigotes and the density of RoR*γ*t^+^, IL-17^+^, IL-1*β*^+^, and caspase-1^+^ cells was observed, but no correlation between Th17 and the inflammasome response with evolutionary disease pathology was reported. These data showed the participation of Th17 cells and the inflammasome in the inflammatory response of the skin lesions of LCL caused by *L.* (*V.*) *panamensis* infection. These results suggest a role in the control of tissue parasitism of IL-17 and the activation of the NLRP3 inflammasome dependent on IL-1*β* but cannot exclude their role in the development of disease pathology.

## 1. Introduction

Cutaneous leishmaniasis (CL) caused by *Leishmania* (*Viannia*) *panamensis* is an endemic disease in Panama [1, 2]. The most prevalent clinical manifestation is ulcerated skin lesions, and a small percentage of patients develop mucosal leishmaniasis (ML) concomitantly with or after CL, leading to tissue damage and occasionally disfiguring facial lesions [1, 3]. Tissue damage in murine and human leishmaniasis has been associated with the exacerbated response of cytokines such as IL-17, IL-1, and TNF*α*, especially in *L.* (*V.*) *braziliensis* infection [4–6]. IL-17 is principally produced by Th17 cells [7, 8], and IL-1 has been related to the differentiation of Th17 cells [9, 10] and inflammasome activation [11–13]. Recently, the participation of Th17 cells and the inflammasome in the immune response against *Leishmania* spp. have been described [6, 12, 14, 15]. Both factors promote the activation of inflammatory processes in infections by *Leishmania* sp. [12, 16, 17], and they seem to have a different role depending on the species of the parasite [13, 15, 16, 18]. Th17 cells play an important role in eliminating pathogens that are not adequately destroyed by Th1 cells [8, 18]. However, Th17 may play an ambiguous role in leishmaniasis since, in visceral disease, they are associated with parasite elimination, and in CL, they are related to the exacerbation of cutaneous lesions and consequently the pathogenesis of the disease [15, 18]. In a murine study with *L.* (*L.*) *major* and in human infection with *L.* (*V.*) *braziliensis*, the effect of Th17 cells was related to the progression of leishmaniasis [5, 6]. In contrast, other studies of *L.* (*V.*) *panamensis* and *L.* (*V.*) *braziliensis* correlated Th17 cells with control and elimination of the parasite [15, 19].

In addition to activation of Th17 cells in response to *Leishmania* sp. infection, we observed activation of the inflammasome that contributes to the inflammatory process at the site of infection. Inflammasomes are multiprotein complexes assembled in the cytoplasm of innate immune cells that regulate the processing of caspase-1 to activate proinflammatory cytokines such as IL-1*β* and IL-18 in response to microbial molecules or stress signals [11, 12, 20, 21]. The NLRP3 inflammasome is the best characterized inflammasome and is composed of the sensor (NLR protein NLRP3) [12, 22], the adapter protein ASC [20], and the inflammatory caspase-1 [11, 23]. Among inflammatory caspases, caspase-1 is the most studied, and its catalytic activity is strongly regulated by the signal-dependent autoactivation of the inflammasome that mediates caspase-1-dependent processing of cytokines, such as IL-1*β* [11]. Several studies have reported that the NLRP3 inflammasome is activated by *Leishmania* spp. and plays an important role in the outcome of the infection [13, 24–27]. The ambiguous role of the inflammasome in the immune response against *Leishmania* sp. has been described [13, 16, 24, 28]. An experimental study in a murine model infected with *L. amazonensis*, *L. braziliensis*, and *L. infantum chagasi* showed that IL-1*β* production derived from the activation of the NLRP3 inflammasome led to host resistance to infection by the production of nitric oxide (NO) [13]. In contrast, activation of the NLRP3 inflammasome and production of IL-1*β* led to an increase in the pathology of murine infection by *L. braziliensis* [16].

Despite the increasing knowledge of the immunopathological mechanisms that contribute to disease progression, the role of Th17 and the inflammasome during *L.* (*V.*) *panamensis* infection remains unclear. Therefore, in this study, we evaluated the Th17 and inflammasome responses in the skin lesions of patients with LCL caused by *L.* (*V.*) *panamensis* to better understand their roles in the immune response against this species of parasite in Panama County, where studies are rare.

## 2. Materials and Methods

### 2.1. Study Design

Samples of skin biopsies (*n* = 46) from patients with positive laboratory and clinical diagnoses of LCL were analysed. The samples were collected at the Clínica de Medicina Tropical of the Instituto Conmemorativo Gorgas de Estudios de la Salud (ICGES), Panama, between January and December 2012. All patients were adults without previous treatment for leishmaniasis and agreed, freely and voluntarily, to participate in the study by providing informed consent. Samples were taken under local anaesthesia and asepsis [29] and analysed by immunohistochemistry at the Laboratorio de Patologia de Moléstias Infecciosas, Universidade de São Paulo, Brazil. *Leishmania* infection was confirmed by direct microscopic observation of amastigotes in Giemsa staining and/or isolation of promastigotes in Schneider's medium from skin scrape samples [29]. The *Leishmania* species was assessed by polymerase chain reaction by kDNA PCR as previously described [30] and characterized as *L.* (*V.*) *panamensis* by PCR-Hsp70/RFLP [31]. After diagnosis, all patients were treated with 20 mg/kg/day of intramuscular glucantime according to the Panamanian guidelines for leishmaniasis control [32].

### 2.2. Ethical Statements

This study was approved by the National Committee of Bioethics of Research of the Gorgas Memorial Institute of Health Studies, Panama, and by the Ethics of Research Committee of the Faculty of Medicine of the University of São Paulo, Brazil, under protocol number 141/13. All the participants signed an informed consent form and voluntarily agreed to participate in the study.

## 3. Sample Collection and Immunohistochemistry Assay

### 3.1. Biopsy Collection

The biopsy samples were taken from the outer edge of the ulcer with a 4 mm Harris punch (Whatman International, Ltd.; UK), followed by the application of local anaesthesia and asepsis [33, 34].

### 3.2. Histopathological Processing

All samples were fixed in 10% buffered formalin and processed within a period of no more than 48 hours to dispose of the paraffin tissue block. All tissue samples were dehydrated, cleared, embedded in paraffin, cut into 4–5 *μ*m thick sections, and prepared for analysis by immunohistochemistry [34–36]. The lesion sections were characterized microscopically based on the histological alterations found in the epidermis and the dermis to correlate with the different markers used to analyse the Th17 and inflammasome responses. The results of the histopathological findings were described in a previous study [37].

### 3.3. Immunohistochemistry

The *in situ* Th17 inflammatory immune response was assessed by immunohistochemistry using anti-IL-6, anti-IL-23, anti-RoR*γ*t, and anti-CD4 monoclonal antibodies and anti-IL-17, anti-TGF-*β*1, and anti-CD3 polyclonal antibodies. The participation of the inflammasome was assessed by immunohistochemistry using anti-IL-1*β*, anti-IL-18, and anti-caspase-1 polyclonal antibodies. Hyperimmune serum from a mouse chronically infected with *L.* (*L.*) *amazonensis* produced in the Laboratory of Pathology of Infectious Diseases was used to confirm tissue parasitism. The histological sections were deparaffinized in xylene for 15 minutes, followed by hydration with a descending series of alcohols. Endogenous peroxidase was blocked with 3% hydrogen peroxide solution (88597, Sigma-Aldrich, USA). Antigen retrieval for the IL-17 and CD4 markers was conducted using 1 mM EDTA buffer at pH 8.0, for the IL-18 marker was conducted with 10 mM Tris/1 mM EDTA at pH 9.0, and for the other markers was conducted with 10 mM citrate buffer at pH 6.0, all in a boiling water bath. Then, primary antibodies were added to the tissues in the following dilutions: anti-*Leishmania* (mouse hyperimmune serum) [38] diluted at 1 : 1000, anti-CD3 (A0452, DakoCytomation, USA), and anti-CD4 (NC-L-CD4-1F6, Novocastra, Leica, USA) diluted at 1 : 50; anti-IL-6 (SC-130326, Santa Cruz Biotechnology, USA) diluted at 1 : 100; anti-TGF-*β*1 (V: SC-146, Santa Cruz Biotechnology, USA) diluted at 1 : 200; anti-IL-17 (H-132: SC-7927, Santa Cruz Biotechnology, USA) diluted at 1 : 200; anti-RoR*γ*t (MABF81, 6F3.1, Sigma-Aldrich, USA) diluted at 1 : 2000; anti-IL-23 (C-3: SC-271279, Santa Cruz Biotechnology, USA) diluted at 1 : 2500; anti-IL-1*β* (ab2105, Abcam, UK) diluted 1 : 300; anti-caspase-1 (G6231-3D2, Sigma-Aldrich, USA) diluted at 1 : 500; and anti-IL-18 (ab68435, Abcam, UK) diluted at 1 : 1500. As a negative control, a solution containing phosphate-buffered saline (PBS) and bovine serum albumin (A9647-BSA, Sigma-Aldrich, St. Louis, MO, USA) with the omission of a primary antibody was used, and human amygdala was employed to standardize the reactions. The slides were incubated in a humidified chamber overnight at 4°C. For all markers, the Novolink kit (RE7280-K-Novovastra, Leica, IL, USA) was used. The chromogenic substrate DAB + H_2_O_2_ (K0690-diaminobenzidine with hydrogen peroxide, DakoCytomation, CO, USA) was added to the tissue, incubated for 5 minutes, and counterstained with eosin (Sigma-Aldrich, St. Louis, MO, USA) for anti-RoR*γ*t antibody and Harris haematoxylin (VWR International, PA, USA) for the other antibodies. Finally, the slides were dehydrated in a series of ascending alcohols and mounted with Permount (SP15-500, Fisher Scientific, Waltham, MA, USA) and glass coverslips. Ten skin samples from healthy adult individuals undergoing plastic surgery without current or previous diagnosis of leishmaniasis or any dermatological infection were included as controls.

### 3.4. Quantitative Analysis of Immunostained Cells

Sequential images were obtained using an optical microscope coupled to the microcomputer, and quantification of immunostained cells was performed using the AxioVision 4.8.2 software (Zeiss, San Diego, CA, USA). The images were obtained in the dermis where the inflammatory infiltrate was observed. Ten microscopic fields of each histological section for the different markers were photographed using a 40x objective. Cells were quantified according to cell morphology and brown immunostaining, and cellular density (number of cells per square millimetre) was determined by the ratio of the immunolabeled cells to the area of each image.

### 3.5. Statistical Analysis

The GraphPad Prism 5.0 software (GraphPad Software, San Diego, CA, USA) was used for the statistical analysis of the results. For analysis of the differences between the groups, a *t* test was performed for data with a Gaussian distribution, and the Mann–Whitney test was used for data with a non-Gaussian distribution. For correlation of different markers, Pearson's correlation test was performed for data with a Gaussian distribution and Spearman's correlation test for data with a non-Gaussian distribution. Graphics were made using the Origin 8.0 programme (OriginLab Corporation, Northampton, MA, USA).

## 4. Results

### 4.1. Patient Profile

Forty-six samples from patients with LCL were analysed. Patients came from areas known to be endemic for leishmaniasis in Panama, and most of them were male (72%). The mean age was 33 years, ranging between 21 and 72 years. The median lesion number was 2, ranging between 1 and 8 lesions, with an average of 35 days for the time of evolution, varying from 10 to 90 days. The majority of patients (65%) had lesions with an evolution time ≤ 30 days. Forty-three percent of the patients had ulcerated lesions. The lesions were distributed mostly in the upper extremities (63%), followed by the lower extremities (18%), face/neck (10%), back (5%), and abdomen (4%). All parasites isolated from the skin lesions were characterized as *L.* (*V.*) *panamensis* by PCR-Hsp70/RFLP.

### 4.2. Evaluation of the *In Situ* Th17 Immune Response

**Figure 1:**
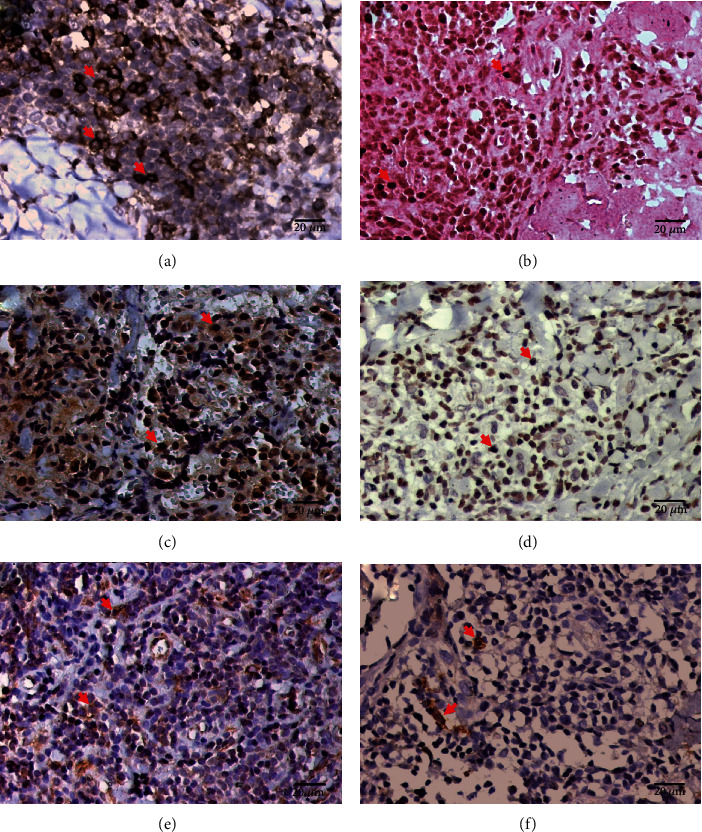
Immunohistochemistry of the skin sections from the patients with LCL; brown CD4^+^ cells (a), RoR*γ*t^+^ cells (b), IL-17^+^ cells (c), IL-23^+^ cells (d), IL-6^+^ cells (e), and TGF-*β*^+^ (f) cells can be observed. The red arrow shows positive cells.

To analyse the Th17 immune response in the skin lesions of the patients with LCL caused by *L.* (*V.*) *panamensis*, we assessed the CD3, CD4, RoR*γ*t, IL-17, IL-6, TGF-*β*, and IL-23 markers by immunohistochemistry (see Figure 1). The cellular densities (mean ± the standard error) of these markers were 2593.00 ± 112.00 cells/mm^2^ for CD3^+^, 914.50 ± 51.76 cells/mm^2^ for CD4^+^, 229.20 ± 13.49 cells/mm^2^ for RoR*γ*t^+^, 859.80 ± 70.66 cells/mm^2^ for IL-17^+^, 132.20 ± 9.50 cells/mm^2^ for TGF-*β*^+^, 273.20 ± 15.89 cells/mm^2^ for IL-6^+^, and 669.80 ± 34.73 cells/mm^2^ for IL-23^+^ (see Table 1). The density of different markers observed in the skin biopsies from individuals infected with *L.* (*V.*) *panamensis* was higher than that observed in healthy skin (*p* < 0.0001) (see Table 1).

Considering the time of infection (≤30 days versus >30 days), gender (male versus female), or histopathological tissue response (granulomas versus nongranuloma reaction and presence versus absence of ulcer), no significant difference was observed between the markers used to analyse the Th17 immune response (*p* > 0.05).

**Figure 2:**
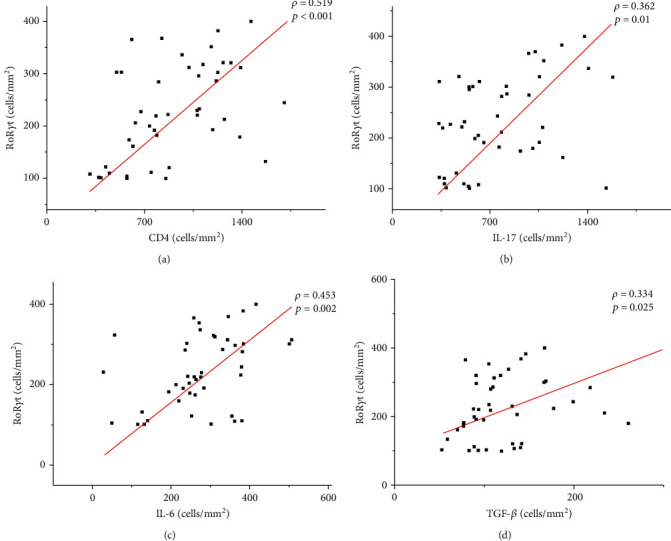
Graphs showing the correlation between the cellular density of RoR*γ*t^+^ cells and CD4^+^ cells (a), IL-17^+^ cells (b), IL-6^+^ cells (c), and TGF-*β*^+^ cells (d). The value of *ρ* is Spearman's correlation coefficient, and *p* is the *p* value. The red line indicates a positive correlation.

**Figure 3:**
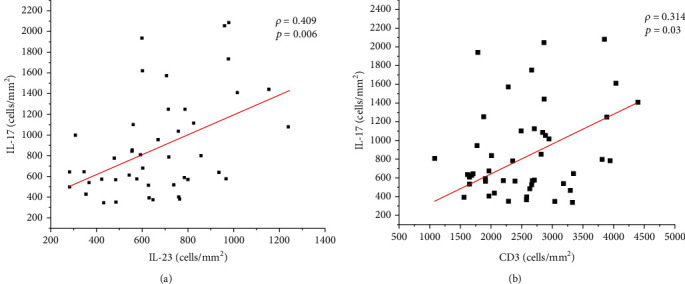
Graphs showing the correlation between the cellular density of IL-17^+^ cells and IL-23^+^ cells (a) and CD3^+^ cells (b). The value of *ρ* is Spearman's correlation coefficient, and *p* is the *p* value. The red line indicates a positive correlation.

**Figure 4:**
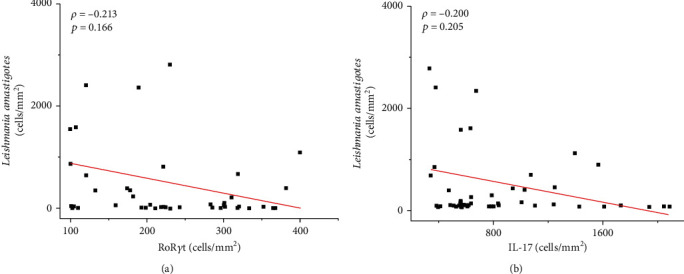
Graph showing the correlation between the density of *Leishmania* amastigotes and RoR*γ*t^+^ cells (a) and IL-17^+^ cells (b). The value of *ρ* is Spearman's correlation coefficient, and *p* is the *p* value. The red line indicates a negative correlation.

According to the linear correlation analysis between the evaluated markers, we observed a positive correlation between RoR*γ*t^+^ and CD4^+^ (moderate: *ρ* = 0.519; *p* < 0.0001), IL-17^+^ (weak: *ρ* = 0.362; *p* = 0.01), IL-6^+^ (weak: *ρ* = 0.453; *p* = 0.002), and TGF-*β*^+^ (weak: *ρ* = 0.334; *p* = 0.02) (see Figure 2). In addition, there was a positive correlation between IL-17^+^ and IL-23^+^ (weak: *ρ* = 0.409; *p* = 0.006) and CD3^+^ (weak: *ρ* = 0.314; *p* = 0.03) (see Figure 3) and a positive correlation between TGF-*β*^+^ and IL-6^+^ (moderate: *ρ* = 0.541; *p* < 0.0001). Although not statistically significant, an inverse correlation was observed between the density of amastigotes and the density of RoR*γ*t^+^ (*ρ* = −0.213; *p* > 0.05) and IL-17^+^ cells (*ρ* = −0.200; *p* > 0.05) (see Figure 4).

### 4.3. Evaluation of Inflammasomes in the *In Situ* Immune Response against *L.* (*V.*) *panamensis*

**Figure 5:**
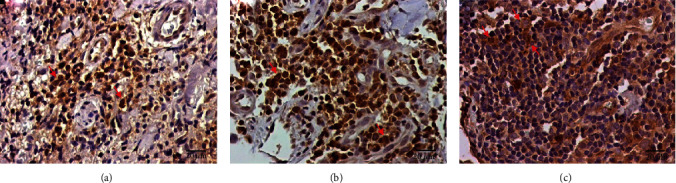
Immunohistochemistry of the skin sections from the patients with LCL showing brown IL-1*β*^+^ cells (a), IL-18^+^ cells (b), and caspase-1^+^ cells (c). The red arrow indicates positive cells.

The markers IL-1*β*, IL-18, and caspase-1 were assessed to evaluate the participation of the canonical NLRP3 inflammasome in the immune response against *L.* (*V.*) *panamensis* infection (see Figure 5). The cellular density (mean ± the standard error) of IL-1*β*^+^ was 645.90 ± 72.33 cells/mm^2^, that of IL-18^+^ was 73.45 ± 8.84 cells/mm^2^ and that of caspase-1^+^ was 485.00 ± 64.17 cells/mm^2^ (see Table 2). The cellular density of these markers was higher in the skin sections of the *L.* (*V.*) *panamensis*-infected patients than the control skin sections (*p* < 0.0001) (see Table 2).

Considering the time of infection (≤30 days versus >30 days), gender (male versus female), and histopathological tissue response (granulomas versus nongranuloma reaction and presence versus absence of ulcer), no significant difference was observed in the cellular densities of IL-1*β*, IL-18, and caspase-1 (*p* > 0.05).

**Figure 6:**
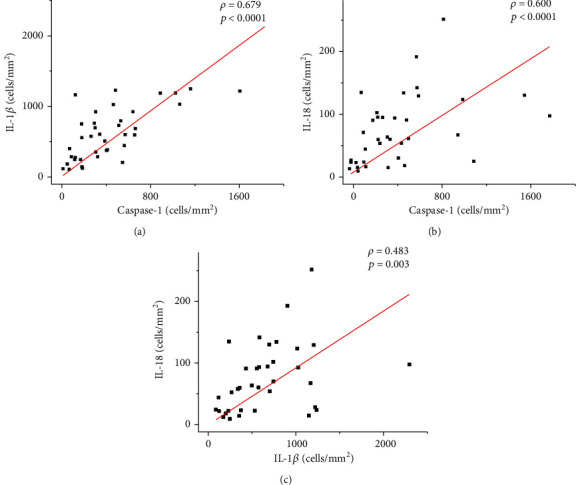
Graphs showing the correlation between the cellular density of Caspase-1^+^ cells with IL-1*β*^+^ cells (a) and IL-18^+^ cells (b). The positive correlation between IL-1*β*^+^ cells and IL-18^+^ cells (c). The value of *ρ* is Spearman's correlation coefficient, and *p* is the *p* value. The red line indicates a positive correlation.

**Figure 7:**
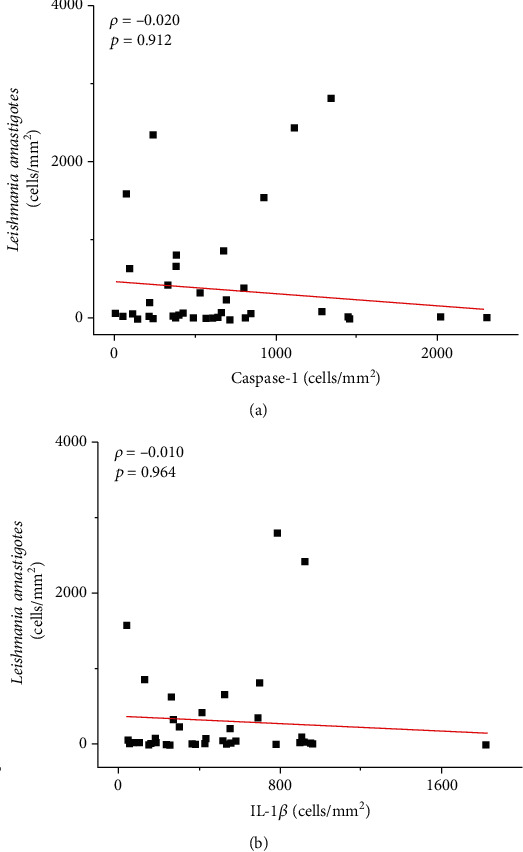
Graphs showing the correlation between the density of *Leishmania* amastigotes and caspase-1^+^ cells (a) and IL-1*β*^+^ cells (b). The value of *ρ* is Spearman's correlation coefficient, and *p* is the *p* value. The red line indicates a negative correlation.

A positive correlation was observed between caspase-1 and IL-1*β* (strong: *ρ* = 0.679; *p* < 0.0001) as well as between caspase-1 and IL-18 (moderate: *ρ* = 0.600; *p* < 0.0001) and between IL-1*β* and IL-18 (weak: *ρ* = 0.483; *p* = 0.003) (see Figure 6). Although not statistically significant, an inverse correlation was observed between the density of amastigotes and the cellular density of IL-1*β*^+^ (*ρ* = −0.020; *p* > 0.05) and caspase-1^+^ cells (*ρ* = −0.010; *p* > 0.05) (see Figure 7).

## 5. Discussion

Recent studies have shown the participation of Th17 cells and the inflammasome in the immune response against *Leishmania* sp. infection. Both factors contribute to the production of proinflammatory cytokines by favouring the inflammatory response at the site of infection [6, 12, 14, 15]. Among the proinflammatory cytokines, IL-1 is related to the differentiation of Th17 cells and the activation of the inflammasome [9–13]. Th17 (RoR*γ*t) cells produce IL-17 [8, 39], whose main function is the induction of tissue inflammation and protection of the host against pathogens [8]. The present study showed the presence of CD4^+^, RoR*γ*t^+^, and IL-17^+^ cells, as evidenced by immunohistochemistry, and the linear correlation analysis of these data showed a positive correlation among these markers, suggesting the presence of Th17 cells (CD4^+^RoR*γ*t^+^) producing IL-17 in the skin biopsies of the patients with LCL caused by *L.* (*V.*) *panamensis*. The cellular density of IL-17^+^ cells was greater than the density of RoR*γ*t^+^ cells, indicating that other cells could produce this cytokine in the cutaneous lesions. According to the literature, most IL-17 produced in the CL is mainly from TCD4^+^RoR*γ*t^+^ cells; however, IL-17 can also be secreted by other cell types, such as TCD8, T*γδ*, and NK cells and monocytes [40]. Therefore, in the present study, we cannot rule out the possibility that other cellular types, such as T*γδ* cells and double-negative CD3 lymphocytes, are producing IL-17 [41].

Correlations between RoR*γ*t^+^ cells and IL-6^+^ and TGF-*β*^+^ cells were observed (*p* < 0.002 and *p* < 0.02, respectively), which suggests the participation of these cytokines in the differentiation of Th17 cells [42–44]. We also observed a weak positive correlation between IL-1*β*^+^ cells and RoR*γ*t^+^ cells (*ρ* = 0.320; *p* = 0.04), suggesting the possible participation of IL-1*β* in the development of Th17 cells, as has been described elsewhere [9, 10]. In addition, there was a correlation between IL-17 and IL-23 (*p* = 0.006), suggesting the important role of IL-23 in the maintenance of IL-17-producing Th17 cells [8, 18]. Although not statistically significant, an inverse correlation was observed between the density of amastigotes and the density of RoR*γ*t^+^ and IL-17^+^ cells, suggesting the control of tissue parasitism as described in murine studies since IL-17 together with IFN-*γ* has been demonstrated to have an important role in the resolution of the disease caused by *Leishmania* (*Viannia*) parasites [15, 19]. However, the presence of IL-17 has also been associated with the worsening of the disease outcome due to the increase in the lesion size and the presence of ulcers [5]. Unfortunately, in the present study, due to the absence of data about lesion size, we were unable to establish these correlations and associate them with disease severity. Despite the presence of ulcers in 43% of the skin biopsies, we did not observe a correlation indicating a detrimental role of Th17 cells, probably due to the presence of other mechanisms or cytokines, such as TNF-*α*, that could be involved in the tissue damage observed in the cutaneous lesions [4, 45]. Notably, the lesions analysed in this study were relatively recent, and the majority of patients (65%) had lesions with an evolution time ≤ 30 days, varying from 10 to 90 days. The results suggest that Th17 cells can help eliminate parasites through IL-17 in the activation of host cells; however, their role in the progression of the disease pathology caused by *L.* (*V.*) *panamensis* cannot be disregarded.

An imbalanced immune response in *L.* (*V.*) *panamensis* infection involving the production of both inflammatory and anti-inflammatory cytokines is responsible for the maintenance of inflammation, which plays an important role in the pathogenesis of leishmaniasis [46, 47]. In this way, other inflammatory cytokines, such as IL-1 and IL-18, that are directly related to inflammasome activation could be part of this process and be directly involved in the skin lesions caused by this species of parasite [12, 13]. The data obtained in our study showed the presence of IL-1*β* and IL-18 cytokines as well as caspase-1, which are canonical markers to assess the presence of NLRP3 inflammasome activation in *Leishmania* (*Viannia*) infection, in the inflammatory response against *L.* (*V.*) *panamensis* [11, 12]. Their presence, evidenced by immunohistochemistry, is correlated with the moderate to intense inflammatory infiltrate observed in the skin lesions of LCL caused by *L.* (*V.*) *panamensis*. Although the activation of the NLRP3 inflammasome and the production of IL-1*β* lead to an increase in the pathology of murine infection by *L. braziliensis* [16], we observed an inverse correlation between the density of amastigotes and the densities of IL-1*β* and caspase-1, suggesting the role of the inflammasome in the control of *L.* (*V.*) *panamensis* infection as has been described previously [13, 28]. Inflammasome activation was shown to be important for the restriction of parasite replication in a murine model of infection induced by *L. amazonensis*, *L. braziliensis*, and *L. infantum chagasi* infection [13]. These species of the *Leishmania* parasite trigger the activation of caspase-1 in macrophages, leading to the production of nitric oxide (NO), which is important for clearance of the parasite. Another *in vitro* study showed that the activation of the NLRP3 inflammasome promotes host resistance against *L. braziliensis* infection through NO production by macrophages [28]. Although these data suggest the important role of the inflammasome in the control of *Leishmania* parasites, its pathogenic role in the infection caused by *L.* (*V.*) *panamensis* cannot be discarded. Further studies regarding the role of inflammasomes in *L.* (*V.*) *panamensis* infection, including correlations with the evolution of infection and mucous involvement, are needed.

## 6. Conclusions

These data suggest the participation of Th17 cells and the inflammasome in the *in situ* inflammatory response in localized cutaneous leishmaniasis caused by *L.* (*V.*) *panamensis* infection and their roles in the control of the parasites, probably through IL-17 and the IL-1*β*-dependent NLRP3 inflammasome activation; however, the results cannot exclude their inflammatory role in the development of disease pathology.

## Figures and Tables

**Table 1:** Mean and standard error of the cellular densities (cells/mm^2^) of the different markers used to evaluate the Th17 immune response in the skin lesions of the patients with localized cutaneous leishmaniasis (LCL) caused by *L.* (*V.*) *panamensis* and in healthy skin.

Antibody	LCL skin (*n* = 46)	Healthy skin (*n* = 10)	*p* value
CD3	2593.00 ± 112.00	37.30 ± 8.13	*p* < 0.0001
CD4	914.50 ± 51.76	46.25 ± 11.55	*p* < 0.0001
RoR*γ*t	229.20 ± 13.49	0.10 ± 0.01	*p* < 0.0001
IL-17	859.80 ± 70.66	18.64 ± 5.03	*p* < 0.0001
IL-6	273.20 ± 15.89	7.31 ± 2.14	*p* < 0.0001
TGF-*β*	132.20 ± 9.50	0.10 ± 0.01	*p* < 0.0001
IL-23	669.80 ± 34.73	0.10 ± 0.01	*p* < 0.0001

**Table 2:** Mean and standard error of the cellular densities (cells/mm^2^) of the different markers used to evaluate the inflammasome response in the skin lesions of the patients with localized cutaneous leishmaniasis (LCL) caused by *L.* (*V.*) *panamensis* and in healthy skin.

Antibody	LCL skin *n* = 46	Healthy skin *n* = 10	*p* value
IL-1*β*	645.90 ± 72.33	0.10 ± 0.01	*p* < 0.0001
IL-18	73.45 ± 8.84	0.10 ± 0.01	*p* < 0.0001
Caspase-1	485.00 ± 64.17	0.10 ± 0.01	*p* < 0.0001

## Data Availability

The datasets generated for this study are available upon request from the corresponding author.
